# Design of a Slab Tamm Plasmon Resonator Coupled to a Multistrip Array Waveguide for the Mid Infrared

**DOI:** 10.3390/s22082968

**Published:** 2022-04-13

**Authors:** Gerald Pühringer, Cristina Consani, Reyhaneh Jannesari, Clement Fleury, Florian Dubois, Jasmin Spettel, Thang Duy Dao, Gerald Stocker, Thomas Grille, Bernhard Jakoby

**Affiliations:** 1 Institute for Microelectronics and Microsensors, Johannes Kepler University, 4040 Linz, Austria; reyhaneh.jannesari@jku.at (R.J.); bernhard.jakoby@jku.at (B.J.); 2 Silicon Austria Labs GmbH, 9524 Villach, Austria; cristina.consani@silicon-austria.com (C.C.); clement.fleury@silicon-austria.com (C.F.); florian.dubois@silicon-austria.com (F.D.); jasmin.spettel@silicon-austria.com (J.S.); thang.dao@silicon-austria.com (T.D.D.); 3 Infineon Technologies Austria AG, 9500 Villach, Austria; gerald.stocker@infineon.com (G.S.); thomas.grille@infineon.com (T.G.)

**Keywords:** silicon photonics, Tamm plasmons, waveguide sensing, mid-infrared sensing

## Abstract

In this work, we present and analyze a design of an absorber–waveguide system combining a highly sensitive waveguide array concept with a resonant selective absorber. The waveguide part is composed of an array of coupled strip waveguides and is therefore called a coupled strip array (CSA). The CSA is then coupled to the end of a slab Tamm plasmon (STP-) resonator, which is composed of a quasicrystal-like reflector formed by the patterning of a silicon slab and an interfacing tungsten slab. The concept describes an emitter–waveguide or waveguide–detector system featuring selective plasmon-enhanced resonant absorption or emission. These are crucial properties for corresponding optical on-chip integrated devices in context with evanescent field absorption sensing in fluids or gases, for example. Thus, the concept comprises a valuable and more cost-effective alternative to quantum cascade lasers. We designed the lateral dimensions of the STP resonator via a simple quasi-crystal approach and achieved strong narrowband resonances (emittance and *Q*-factors up to 85% and 88, respectively) for different silicon thicknesses and substrate materials (air and silicon oxide). Moreover, we analyze and discuss the sensitivity of the complete emitter–waveguide system in dependence on the slab thickness. This reveals the crucial correlation between the expected sensitivity assigned to the absorber–waveguide system and field confinement within the silicon.

## 1. Introduction

The integration of efficient detectors or thermal sources into on-chip sensing systems is strongly desired by the industry, as they have the potential to offer a cost-effective alternative to current mid-infrared solutions involving quantum cascade structures [1]. One of the many approaches are Tamm plasmon–polariton (TP) absorbers and emitters [2,3,4,5,6]. We recently proposed the concept of a slab Tamm plasmon (STP) absorber, which features inherent coupling to (and from) the fundamental mode of a slab waveguide [7,8]. Such a structure constitutes a complete integrated waveguide–absorber system (WAS), which has high potential for various waveguide sensing applications. In the present paper, we combine the STP resonators with the more efficient coupled-strip array (CSA) waveguide concept in view of evanescent wave absorption sensing. A CSA supports a guided quasi-TE mode featuring a significant fraction of the field in the sensing region outside the silicon, as characterized by a larger external confinement factor Γ (for exact definition see [9,10], for example) while preserving high propagation length (or low wave attenuation) simultaneously. These two quantities are essential for the sensitivity of waveguides used in integrated optical absorption sensing [11]. Consequently, we evaluate the WAS via a corresponding Figure of Merit reflecting the sensitivity of the structure in context with bulk or surface absorption sensing. As we aim for wavelength-selective emission in context with CO_2_ sensing, the target resonance wavelength $\lambda_{r}$ is chosen to be 4.26 μm (asymmetric stretching vibration absorption line). The CSA waveguides (without the resonator part) were already successfully fabricated and experimentally tested for the corresponding wavelength range [12].

In principle, we demonstrate strong coupling between a sensitive waveguide mode and a strong STP resonance. The STP resonator features the quasicrystal-like patterning of silicon and tungsten as plasmonic material in a geometrical arrangement, which has high potential to be compatible with standard semiconductor processes. Correspondingly, the height *h* of the Si slab is assumed to be constant throughout the structure. As a result, such a WAS concept enables monolithic integration via a cost-effective fabrication processes. The selective resonant absorption can be utilized for the detection of mid-IR radiation (by means of thermopiles, for example) or, by virtue of Kirchhoff’s law, as a resonant selective emitter [7,13]. Resonant wavelengths adjacent to $\lambda_{r}$ (within the photonic stopband) become suppressed by the resonator [14]. The spectral response is expected to be highly polarization dependent, as the reflective properties of metal surfaces change drastically if the spatial extent is limited in one dimension. This property is exploited by wire-grid polarizers, for example, but was also studied in context with TP structures [15,16]. The consequence for STP structures is that only the fundamental quasi-TE multistrip waveguide mode can be coupled to the resonator. Thus, other waveguide modes (i.e., quasi-TM or higher order modes) cannot be coupled to the TE-polarized STP resonance [7]. This should lead to a corresponding non-resonant broadband thermal background emission for the operation of the WAS as an emitter.

**Figure 1:**
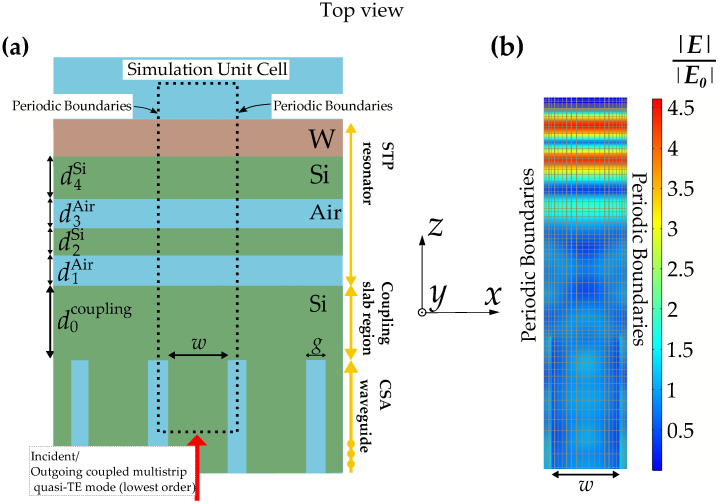
(**a**) Top view of a slab Tamm-plasmon WAS with tungsten as plasmonic material. The simulation unit cell is indicated by the dotted rectangle. (**b**) Corresponding normalized electric field enhancement at $\lambda_{r}$. The assumed incident guided mode is (quasi) TE polarized (lowest order, electric field mainly along *x*-axis).

**Figure 2:**
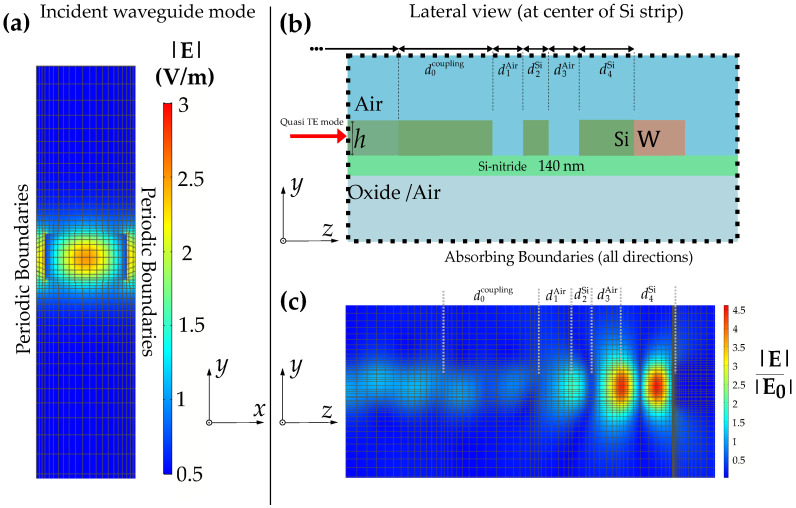
(**a**) Electric field profile with h= 800 nm and air as a substrate for the incident quasi-TE mode (lowest order) of the coupled strip obtained from boundary mode analysis. (**b**) Illustration of the layer stack showing the lateral cross-section at the slab center. (**c**) Same cross-section as (**b**) but showing the profile of the electric field enhancement. The field profiles show the mesh elements from the corresponding perspective. Note the high mesh density at the Si–W interface for accurate evaluation of the power absorbed by the metal.

The system is composed of three parts (see Figure 1a and Figure 2b, see yellow arrows): the CSA waveguide, the STP resonator and a region of a continuous Si slab in between (slab coupling region). The structure is modeled and simulated using a finite element method (FEM) software package (COMSOL Multiphysics 5.5). The lateral periodicity enables efficient simulations via periodic boundary conditions.

## 2. Design of Waveguide and Resonator Structure

### 2.1. Coupled Strip Array Waveguide

The geometric dimensions of the waveguide unit cell are depicted in Figure 1a and Figure 2b (strip width *w*, gap width *g*, slab thickness *h*). These parameters determine the trade-off between the confinement factor Γ and the waveguide base attenuation coefficient α [11]. The former is a measure of the interaction with the analyte, whereas the latter characterizes the attenuation of the waveguide mode without sensing medium. We consider α to be exclusively determined by the dielectric losses (i.e., no surface roughness or other scattering effects are considered in the simulation model). Therefore, the experimental assessment of the waveguide is crucial to estimate the WAS sensitivity. Successful fabrication and experimental evaluation of α and Γ was reported in [12] for the CSA–waveguide geometry featuring g=0.3 µm, w=1.3 µm and h=0.66 µm. The measured values of Γ≈16% and α≈1.3 cm${}^{-1}$ agreed reasonably well with corresponding simulations yielding Γ≈18% and α≈0.6 cm${}^{-1}$. These simulations assumed that the dampening originates exclusively from the oxide [17], which explains the the lower value for α obtained from the simulations.

However, as α is highly dependent on the intrinsic absorption properties of all silicon-based materials involved (including polysilicon or silicon nitride), small variations of the imaginary part of the refractive index $n^{{}_{{}^{\prime \prime }}}$ (or extinction coefficient) can lead to drastic changes of α, whereas Γ is more robust with respect to those variations. Literature values of $n^{{}_{{}^{\prime \prime }}}$ at mid-IR wavelengths are generally not reliable for silicon-based materials due to their small magnitude and the variability of on-chip silicon variants, particularly for polysilicon and silicon-nitride [18]. As a result, it is reasonable to set the extinction coefficients for polysilicon and silicon-nitride ($n_{\mathrm{Si}}^{{}_{{}^{\prime \prime }}}$ and $n_{\mathrm{SiN}}^{{}_{{}^{\prime \prime }}}$, respectively) to zero for the simulations in [12].

The aforementioned agreement between the simulation and experiment for the values of α and Γ can be improved by assuming the same $n^{{}_{{}^{\prime \prime }}}$ for nitride as for oxide, namely $n_{{\mathrm{SiO}}_{2}}^{{}_{{}^{\prime \prime }}}=n_{\mathrm{SiN}}^{{}_{{}^{\prime \prime }}}∼4.3\times 10^{-4}$ (from [17]). Then, the simulation yields the values Γ=18% and α=1.33 cm${}^{-1}$, which are very close to the experiments.

The extinction coefficients $n^{{}_{{}^{\prime \prime }}}$ (in principle for all materials) depend on material imperfections such as defects, grain size or residual materials. In addition, the previous study by Ranacher et al. [18] even suggests a slightly higher single-strip waveguide damping with the thin silicon-nitride membrane.

For this reason, the assumption $n_{{\mathrm{SiO}}_{2}}^{{}_{{}^{\prime \prime }}}=n_{\mathrm{SiN}}^{{}_{{}^{\prime \prime }}}$ seems to be a better base for the evaluations in this study. Consequently, the corresponding material dispersions of the extinction coefficients are also set to be equal for the simulations and evaluations of the total WAS.

Nevertheless, it should be kept in mind that these simulations only provide a platform for optimization principles, as an effective extinction coefficient may not cover all waveguide damping mechanisms accurately.

For this study, we increase the width of the strip *w* to 1.4 µm and keep the gap between two strips *g* at 0.3 µm and the target wavelength to 4.26 µm without loss of generality. The WAS is characterized for four different Si slab thicknesses *h* from 0.7 to 1.1 µm in 0.1 µm steps. The value of *h* is crucial for the light confinement and, subsequently, for α and Γ, characterizing the performance of the waveguide. The aforementioned values meet the requirement of resulting in aspect ratios that are potentially suitable for corresponding standard fabrication processes for micro-electro-mechanical systems (MEMS). In particular, different values for field confinement crucially impact the performance of the resonator (see next section).

The electric field of the incident wave together with the mesh of finite elements obtained via a boundary mode analysis is shown in Figure 2a. It should be mentioned that the waveguide also supports higher order and/or quasi TM modes, especially for thicknesses above 800 nm. However, these modes cannot be in resonance with the adjacent STP resonator. Thus, the STP resonator (described in the next section) inherently enables coupling to (and from) the lowest order quasi-TE mode.

The slightly changed parameters values for *w*, *g* and *h* = 0.7 µm lead to CSA waveguide feature values for Γ (∼14%). This compares to a corresponding single-strip WG (∼10%) or pure slab WG (2%) [18,19].

### 2.2. Slab Tamm Plasmon Resonator/Quasi-Crystal Approach

For the design of the STP resonator, we followed a quasi-crystalline patterning approach. Thus, the individual thicknesses were arranged in terms of multiples of quarter-wavelength layers. In the case of conventional TP structures (featuring infinitely extended layer of alternating optical density), those are dimensioned as λ/4n (for light with perpendicular incidence), where *n* denotes the refractive index of each corresponding layer. As for STP structures, we use the effective index of the corresponding fundamental slab waveguide mode (TE) instead. In analogy to the heuristic approach in [8], we design the Si regions of the resonator as $d_{2}^{\mathrm{Si}}=\lambda_{r}/4n_{\mathrm{eff}}+\delta_{\mathrm{skin}}n_{\mathrm{eff}}$ and $d_{4}^{\mathrm{Si}}=3\lambda_{r}/4n_{\mathrm{eff}}+\delta_{\mathrm{skin}}n_{W}$ (see Figure 1a), where $n_{\mathrm{eff}}$ is the effective refractive index of the fundamental TE slab waveguide mode, $\delta_{\mathrm{skin}}=2\pi \kappa_{W}/\lambda_{r}$ is the penetration depth of the field into tungsten and $n_{W}+i\kappa_{W}$ is the complex refractive index of tungsten at $\lambda_{r}$. The air regions $d_{1}^{\mathrm{Air}}$ and $d_{3}^{\mathrm{Air}}$ are both dimensioned as $\lambda_{r}/8$, such that the optical path length of both gaps corresponds to a single quarter-wavelength layer. The electric field distribution at resonance together with the mesh of finite elements shown in Figure 1b and Figure 2c allows to spot quasi-crystal composition.

The width of the tungsten (in z-direction) is constant for all configurations and arbitrarily set to 1 µm. There is only a lower limit (∼300 nm) to this thickness to avoid transmission through the metal or thin film effects (valid for all STP structures). Thus, there is no corresponding upper limit (from a wave-optics point-of-view), which may be particularly interesting for the device fabrication.

### 2.3. Dimensioning of Coupling Region

The intermediate region between the CSA WG and the STP resonator is supposed to couple the WG mode to the STP mode, thus ideally acting as an anti-reflective layer. Therefore, the resulting field in this region can be regarded as a superposition between the slab mode and the coupled multistrip waveguide mode. Thus, we replace $n_{\mathrm{eff}}$ by a superposition of n${}_{\mathrm{eff}}$ and $n_{\mathrm{eff}}^{\mathrm{CSA}}$, where $n_{\mathrm{eff}}^{\mathrm{CSA}}$ is the effective index of the coupled multistrip waveguide mode. As a first heuristic approach, we take the arithmetic mean of these two values. Thus, the slab coupling region is dimensioned as $d_{0}^{\mathrm{coupling}}=\frac{5\lambda }{4n_{\mathrm{eff}}^{\mathrm{avg}}}+\delta_{\mathrm{skin}}n_{\mathrm{eff}}^{\mathrm{avg}}$ with $n_{\mathrm{eff}}^{\mathrm{avg}}=\left(n_{\mathrm{eff}}+n_{\mathrm{eff}}^{\mathrm{CSA}}\right)/2$. It can be seen in Figure 1b that the electric field distribution in the coupling region is non-uniform in the lateral (x-) direction, despite a pure slab geometry (i.e., no gap).

Table 1 shows all the dimensions resulting from the quasi-crystalline patterning approach for each slab thickness *h*. All structures feature the same values for *g* and *w*. Theoretically, the dimension differs slightly between the configurations with and without oxide as substrate material as a result of the slightly varying effective indices. However, these differences are in the order of magnitude of nanometers and are neglected for Table 1.

## 3. FOM of Emitter–Waveguide System

The performance of a specific waveguide type in terms of sensitivity is generally related to the smallest detectable change in analyte concentration [11,20]. This leads to an expression for the sensitivity and, subsequently, to a Figure-of-Merit (FOM) as a function of α and of Γ the form

(1) $$\begin{array}{c} {\mathrm{FOM}}_{\mathrm{WG}}=\frac{\alpha_{0}\Gamma }{\alpha \lambda_{r}}\;, \end{array}$$

where $\alpha_{0}$ denotes the bulk absorption coefficient of the analyte (including the molecular absorption and the concentration of the analyte). We note here that Equation (1) is valid for an optimal length of maximum sensitivity $z_{\max}∼1/\alpha$ [11], which implies very low concentrations of the sensing analyte (CO${}_{2}$). The value of $z_{\max}$ for the CSA waveguides investigated in the present paper is on the order of magnitude of centimeters, which renders them feasible for on-chip integration. The bulk absorption coefficient $\alpha_{0}$ is set to unity for the following discussion, as Equation (1) should be independent of any specific analyte concentration (change). As mentioned previously, in this simulation, the value for α originates exclusively from the damping associated with absorption in the silicon oxide and nitride.

**Figure 3:**
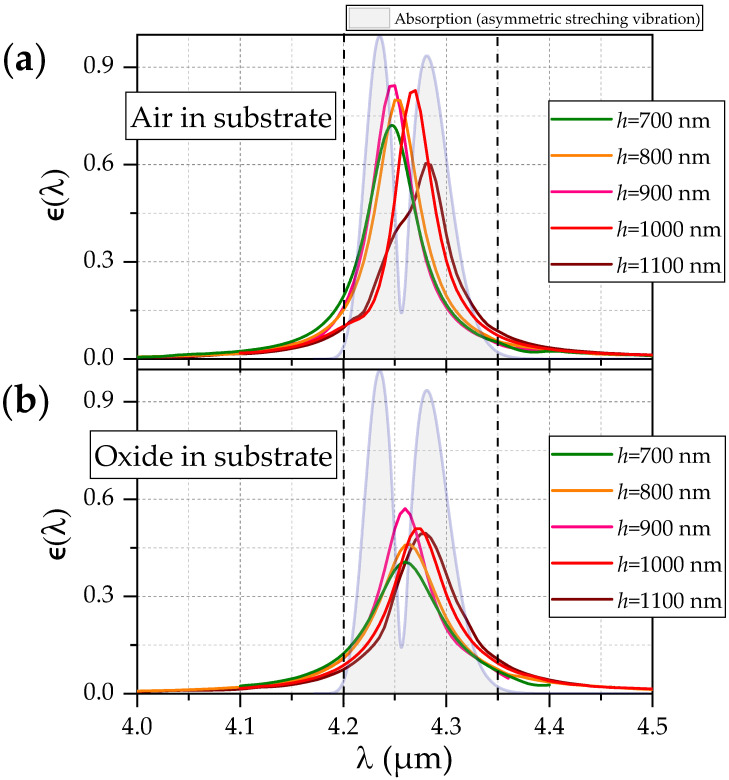
(**a**) Spectral response (i.e., power absorbed by W or, by Kirchhoff’s law, the emittance ϵ(λ)) of the resonator structure with air as substrate material. The shaded graph indicates the normalized CO${}_{2}$ absorption line obtained from [21] (**b**) Analogous to (**a**) but now with SiO${}_{2}$ as material for the substrate.

We now include the selective absorption or emission of the resonator into this FOM. The calculated absorptance a(λ) (which equals the emittance ϵ(λ) by Kirchhoff’s law) obtained by calculating the net power flux between the Si–W interface via integrating the Poynting vector—see [7]—of the STP resonator allows us to estimate the resulting radiation power via Planck’ s law as $P_{\epsilon }\left(T\right)=\int_{\lambda_{1}}^{\lambda_{2}}\epsilon \left(\lambda \right)p_{\mathrm{BB}}\left(\lambda ,T\right)d\lambda$, where $p_{\mathrm{BB}}\left(\lambda ,T\right)$ denotes the power spectrum of an ideal blackbody source. The integration boundaries correspond to the assumed constant absorption bandwidth $\Delta \lambda^{{\mathrm{CO}}_{2}}=\lambda_{2}-\lambda_{1}$. In the case of the target CO${}_{2}$ absorption band, these bounds $\lambda_{1}$ and $\lambda_{2}$ are chosen according to the width of the absorption band (4.2 and 4.35 µm, respectively; see vertical dashed lines in Figure 3).

It should be noted that ϵ(λ) is temperature dependent in general, as the materials (the dielectrics as well as the metal) feature temperature-dependent optical properties. In this work, we consider all materials involved to be at ambient temperature, as then the optical properties can be conveniently taken from literature in this case. Previous studies have shown that strong TP resonances can also be achieved for higher material temperatures [2,22]. In order to significantly reduce the temperature dependence of the emitted power, we use the normalized power

(2) $$\begin{array}{c} p_{\epsilon }\left(T\right)=\frac{P_{\epsilon }\left(T\right)}{\int_{\lambda_{1}}^{\lambda_{2}}p_{\mathrm{BB}}\left(\lambda ,T\right)d\lambda }=\frac{\int_{\lambda_{1}}^{\lambda_{2}}\epsilon \left(\lambda \right)\cdot p_{\mathrm{BB}}\left(\lambda ,T\right)d\lambda }{\int_{\lambda_{1}}^{\lambda_{2}}p_{\mathrm{BB}}\left(\lambda ,T\right)d\lambda } \end{array}$$

instead of $P_{\epsilon }\left(T\right)$ for a more general FOM of the complete resonator–waveguide system. Equation (2) is only weakly dependent on T, particularly for higher temperatures. Here, we assume an operation temperature $T_{O}=$ 800 K (typical mid-IR operating temperature).

The derivation of Equation (1) (see [11], for example), is based on the derivative of the fractional optical power change (P(0)−P(z))/P(0)=ΔP(z)/P(0) with respect to the molecular concentration *m*, i.e., $\frac{d}{dm}\left(\frac{\Delta P(z)}{P(0)}\right)$. Considering now the *absolute* optical power change ΔP(z) instead of the *fractional* optical power change by multiplying the sensitivity figure with the initial intensity P(0) leads to a straight-forward inclusion of P(0) (and, by choice, also of the less temperature dependent version $p_{\epsilon }$) into Equation (1). As a result, the final FOM can be written as a function of the thickness of the silicon slab *h*:

(3) $$\begin{array}{c} {\mathrm{FOM}}_{\mathrm{WG}+\mathrm{emitter}}\left(h\right)=\frac{p_{\epsilon }\left(T_{O},h\right)\cdot \Gamma \left(h\right)}{\alpha \left(h\right)\cdot \lambda_{r}} \end{array}$$

With this definition, the whole spectral window of the absorption line can be exploited to define a measure of the emitted power. It has to be emphasized that Equation (3) can only represent the sensitivity in context with evanescent field (bulk) absorption sensing. In the case of an application of the WAS concept in context with refractive index sensing, the quality factor $Q=\lambda_{r}/\Delta \lambda_{\mathrm{FWHM}}^{\mathrm{source}}$ plays a more dominant role [23].

As shown in our previous works, the thickness of the Si slab *h* correlates strongly with the level of light confinement for STP resonators [7,8] and, subsequently, the peak emittance $\epsilon (\lambda_{r})$ as well as *Q*. By Equation (2), $p_{\epsilon }$ is strongly impacted by *h* as well. At the same time, α will decrease as *h* increases, as crystalline Si can be considered lossless in the mid-IR region. The changes of α and $p_{\epsilon }$ for increasing *h* both result in a higher value of Equation (3). On the other hand, the external confinement factor Γ will decrease, which lowers the value of Equation (3). The total impact of varying *h* is discussed in the next section.

## 4. Results and Discussion

### 4.1. Air in Substrate Region

We first show the spectral response of the waveguide–resonator system, when the all Si structures are solely funded on a thin silicon nitride membrane of 140 nm thickness. Such a configuration provides an extremely high degree of light confinement due to strong refractive index contrast. As can be seen from Figure 3a and Table 2, we observe high absorptances for values of ϵ up to 0.88 for *h* = 0.9 µm and only slightly lower values for *h* = 0.7 and 0.8 µm. Interestingly, we observe a red shift from $\lambda_{r}$ = 4.25 to 4.28 µm together with a slight drop of peak emittance when increasing *h* to 1 and 1.1 µm. It has to be pointed out that the quasi-crystal approach for dimensioning the STP resonators is able to yield resonances almost exactly at the target desired wavelength $\lambda_{r}$ = 4.26 µm without the necessity of scaling. This is despite the heuristic dimensioning approach for the slab coupling region using the averaged effective index $n_{\mathrm{eff}}^{\mathrm{avg}}$.

The spectrum for the structure with h=1100 nm features a shoulder at ∼4.25 μm and a peak at 4.29 μm. This indicates two overlapping peaks. The shoulder can be assigned to an additional parasitic resonance in close spectral vicinity to the main resonance. It emerges due to the rather large slab thickness together with the extent of $d_{0}^{\mathrm{coupling}}$.

**Figure 4:**
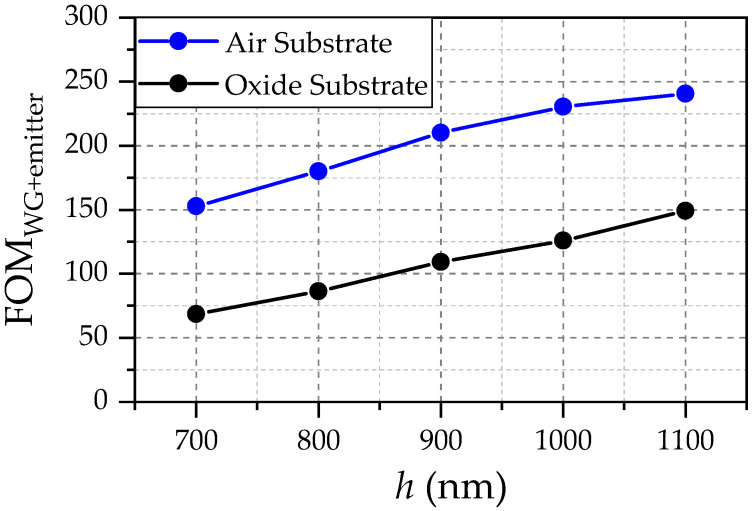
Figure of Merit reflecting the sensitivity of the total WAS different values as a function of the Si slab thickness *h*.

In addition, the *Q* factors are remarkably high when compared with conventional TP structures featuring tungsten in previous studies [2]. The values for the FOM (3) (the sensitivity) increase monotonically with increasing *h*, despite the drop of the peak absorptance (or, equivalently, emittance $\epsilon (\lambda_{r})$) for h>900 nm. This reflects the dominance of the reduced waveguide damping due to increasing confinement within the silicon (and mitigating the damping of the nitride) as can be seen in Figure 4 (black solid line).

For optimal results for the FOM (3), the implementation of an efficient optimization procedure (e.g., genetic algorithms, Bayesian optimization or Monte Carlo Tree search [24,25]) should be able to take full advantage of the higher field confinement for thicker slabs and lead to even higher values for $\epsilon (\lambda_{r})$.

### 4.2. Oxide in Substrate Region

The realization of a thin, suspended nitride membrane carrying all the structures is a great challenge for fabrication. Thus, replacing the air (RI = 1) with silicon oxide as substrate medium may offer a more feasible scenario for fabrication. Figure 3b shows a significant drop in peak emittance as well as in the *Q* factor compared to the suspended structure. The relative drop in these quantities is more pronounced for the lower values of *h*. The latter effect clearly originates from the lower field confinement that thinner slabs can provide in combination with the presence of the substrate, which results in a stronger resonance damping and lower *Q*.

**Figure 5:**
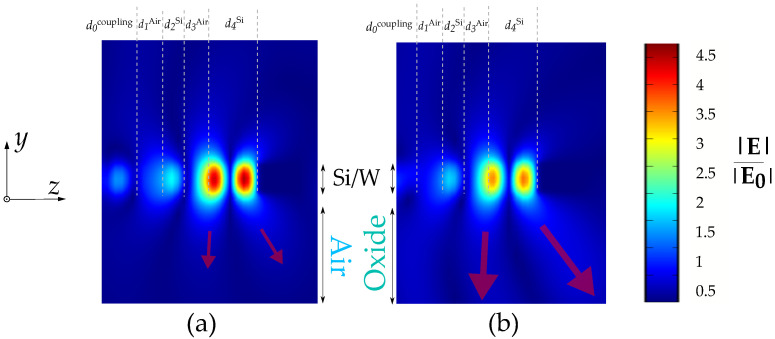
Comparing the electric field profiles between the two configurations featuring (**a**) air in the substrate and (**b**) oxide in the substrate. Both feature the lowest slab thickness h=700 nm. The transparent red arrows indicate the direction and the magnitude of the radiative power loss into the substrate.

This is illustrated by comparing the electric fields in the two panels in Figure 5. In panel (b), an enhanced electric field can be observed, indicating the increased radiation losses into the substrate compared to panel (a). In addition, the plasmonic field enhancement within the Si cavity is reduced in panel (b) as a result of these increased losses. Clearly, panel (b) features oxide as substrate material, whereas panel (a) features air in this region. Both structures feature the slab thickness h=700 nm.

For increasing *h*, the impact of the oxide is slightly less pronounced due to the increased light confinement within the Si.

The FOM shown in Figure 4 reflects the significantly lower level of sensitivity if oxide is present in the substrate.

The reduced value for the peak emittance prohibits better values for the FOM (3) for the thickest slab of 1.1 µm thickness. Although Figure 4 suggests that increasing *h* strictly improves the sensitivity, there are substantial impairments that go along with increasing the Si thickness: first of all, the fabrication becomes significantly more challenging (etching of gaps). In addition, the FOM (3) assumes a waveguide with optimal length according to Γ and α. For the CSA WG, this means that the optimal length is increasing simultaneously with increasing *h*, as the strong reduction in waveguide attenuation is the main reason for the increase we observe in Figure 4. Thus, increasing *h* results in an increased footprint of the device.

The behavior of the sensitivity would substantially differ from the monotone incline if an intrinsic damping mechanism were introduced for the silicon. Then, an optimum value for *h* has to exist, which yields optimal values for Γ, α and $p_{\epsilon }$, maximizing the FOM (3). However, experiments yielded excellent waveguiding properties (i.e., negligible damping) for the polysilicon currently in use, which makes the introduction of additional intrinsic Si-damping not necessary [12].

## 5. Conclusions

We demonstrated the existence of pronounced resonances for the proposed waveguide–resonator system, particularly for the suspended structure. Applying oxide as substrate material resulted in a significant drop in peak emittance and *Q*-factor, reflecting the sensitivity of the STP resonance in regard to the change of the refractive index contrast at the site of the field enhancement. The quasi-crystal approach yields particularly improved results in terms of field enhancement for increasing slab thicknesses up to *h* = 900 nm.

For values of *h* beyond 900 nm, the simple quasi-crystal approach results in a drop of the peak-emittance $\epsilon (\lambda_{r})$. Therefore, a suitable optimization procedure has to be applied here in order to take full advantage of the higher field confinement in the silicon (i.e., reduced radiation losses).

Nevertheless, the unperturbed waveguide (i.e., crystalline silicon, no surface roughness and imperfections) features a monotonically increasing sensitivity reflected by the FOM${}_{\mathrm{WG}+\mathrm{emitter}}$ (3) with increasing *h*, despite the decreasing Γ. Particularly, the continuing increase of Equation (3) for h>900 nm despite decreasing $\epsilon (\lambda_{r})$ has to be highlighted. Thus, the absence of an intrinsic damping mechanism of silicon results in the fact that α is the dominating effect for the waveguide sensitivity (independent from the substrate). The monotone incline of the FOM${}_{\mathrm{WG}+\mathrm{emitter}}$ (3) in Figure 4 would be even more significant for optimized STP–resonator configurations that can take advantage of the higher field confinement of larger slab thicknesses *h*. This monotonically increasing behavior of the sensitivity (i.e., of FOM${}_{\mathrm{WG}}$ (1) as well as of FOM${}_{\mathrm{WG}+\mathrm{emitter}}$ (3)) should not change when considering surface roughness (e.g., the volume-current method [11]), as roughness effects loose significance for increasing field confinement.

In conclusion, this study suggests that if a sufficient quality of the silicon (i.e., negligible intrinsic damping) can be provided, the thickness *h* can be increased for maximum sensitivity as much as the MEMS fabrication process is still feasible for the presented waveguide–absorber system. In addition, the measure for the sensitivity (Equation (3)) is valid for an optimal waveguide length of $z_{\max}∼1/\alpha$, so the waveguides featuring very low α (and, thus, low Γ) demand a larger on-chip footprint. These factors clearly set the limit for the maximum *h* achievable.

Naturally, there are many degrees of freedom for optimization regarding the waveguide (i.e., the parameters *w* and *g*) together with the STP resonator (i.e., the parameters $d_{i}$). In addition, combination with other or additional photonic components such as subwavelength gratings, photonic crystals, (single-) slot waveguides or ring resonators may be able to boost the sensitivity. Nevertheless, we presented a a monolithic optimizable platform of an WAS that could be a valuable part towards a fully integrated mid-infrared on-chip absorption sensor.

## Figures and Tables

**Table 1:** Dimensions in µm for each slab thickness *h*. For each structure, the parameters g=0.3 µm (gap-width) and w=1.4 µm (strip width) were kept constant.

µm					
h	$d_{0}^{\mathrm{coupling}}$	$d_{1}^{\mathrm{Air}}$	$d_{2}^{\mathrm{Si}}$	$d_{3}^{\mathrm{Air}}$	$d_{4}^{\mathrm{Si}}$
0.7	2.065	0.55	0.43	0.55	1.14
0.8	2.01	0.55	0.42	0.55	1.11
0.9	1.96	0.55	0.41	0.55	1.09
1.0	1.92	0.55	0.40	0.55	1.07
1.1	1.89	0.55	0.40	0.55	1.06

**Table 2:** External confinement factors, damping coefficients of the CSA waveguides and plasmon-enhanced absorptance (emittance) and *Q*-factors obtained for the complete waveguide–absorber system.

Substrate Material	*h* (µm)	Γ	α (rad/cm)	$\epsilon (\lambda_{r})$	*Q*
Air	0.7	14.1%	0.72	75%	77
Air	0.8	12.5%	0.55	80%	81
Air	0.9	11.3%	0.43	85%	88
Air	1.0	10.4%	0.34	83%	94
Air	1.1	9.8%	0.28	60.5%	64
Oxide	0.7	13.8%	1.11	40.7%	52
Oxide	0.8	12.3%	0.83	46.1%	58
Oxide	0.9	11.2%	0.64	51.1%	74
Oxide	1.0	10.3%	0.50	57.1%	65
Oxide	1.1	9.7%	0.40	49.6%	60

## Data Availability

Data underlying the results presented in this paper are not publicly available at this time but may be obtained from the authors upon reasonable request.
